# Proteostasis regulation of GABA_A_ receptors in neuronal function and disease

**DOI:** 10.1016/j.biopha.2025.117992

**Published:** 2025-03-20

**Authors:** Xi Chen, Ya-Juan Wang, Ting-Wei Mu

**Affiliations:** Department of Physiology and Biophysics, Case Western Reserve University School of Medicine, Cleveland, OH 44106, USA

**Keywords:** GABA_A_ receptors, Proteostasis, Variants, Epilepsy, Pharmacological Chaperones, Proteostasis regulators, Hispidulin (PubChem CID: 5281628), TP003 (PubChem CID: 10001434), SAHA (PubChem CID: 5311), Verapamil (PubChem CID: 2520), BIX (PubChem CID: 16656807), Dinoprost (PubChem CID: 5280363), Dihydroergocristine (PubChem CID: 107715), AA147 (PubChem CID: 882909), AA263 (PubChem CID: 135509553), 4-phenylbutyric acid (PubChem CID: 4775)

## Abstract

The γ-aminobutyric acid type A receptors (GABA_A_Rs) are ligand-gated anion channels that mediate fast inhibitory neurotransmission in the mammalian central nervous system. GABA_A_Rs form heteropentameric assemblies comprising two α1, two β2, and one γ2 subunits as the most common subtype in mammalian brains. Proteostasis regulation of GABA_A_Rs involves subunit folding within the endoplasmic reticulum, assembling into heteropentamers, receptor trafficking to the cell surface, and degradation of terminally misfolded subunits. As GABA_A_Rs are surface proteins, their trafficking to the plasma membrane is critical for proper receptor function. Thus, variants in the genes encoding GABA_A_Rs that disrupt proteostasis result in various neurodevelopmental disorders, ranging from intellectual disability to idiopathic generalized epilepsy. This review summarizes recent progress about how the proteostasis network regulates protein folding, assembly, degradation, trafficking, and synaptic clustering of GABA_A_Rs. Additionally, emerging pharmacological approaches that restore proteostasis of pathogenic GABA_A_R variants are presented, providing a promising strategy to treat related neurological diseases.

## 1. Introduction

Fast synaptic inhibition in the central nervous system (CNS) is largely mediated by inhibitory neurotransmitters γ-aminobutyric acid (GABA). GABA acts on two types of receptors: the fast-acting, ligand-gated ionotropic GABA_A_ receptors (GABA_A_Rs), and the slower-acting, G-protein coupled metabotropic GABA_B_ receptors (GABA_B_Rs)[1]. GABA_A_Rs are widely distributed across many regions of the mammalian CNS and play an essential role in maintaining the excitatory-inhibitory balance. They belong to the Cys-loop ligand-gated ion channel superfamily that also includes nicotinic acetylcholine receptors (nAChRs), 5-hydroxytryptamine type-3 receptors (5-HT_3_Rs), and glycine receptors (GlyRs)[2]. These receptors consist of five homomeric or heteromeric subunits that surround a central ion-conducting pore. The signature Cys-loop, a conserved loop between two cysteine residues that are separated by 13 residues and covalently linked by a disulfide bond, is located in the large extracellular N-terminal domain of each subunit and plays a critical role in protein folding, subunit assembly, structural integrity and gating of the ion channel[3-5]. GABA_A_Rs are heteropentameric GABA-gated chloride channels. To date, a total of 19 subunit genes that encode GABA_A_Rs have been identified in the human brain, which are divided into subsequent classes based on sequence similarities: α1-α6, β1-β3, γ1-γ3, δ, ε, π, θ, and ρ1-ρ3 [6]. The distribution of GABA_A_R subunits varies across different brain regions, including the cortex, hippocampus, and cerebellum, which contributes to the vast heterogeneity and functional diversity of GABA_A_Rs. For example, while α1, β1, β2, β3, and γ2 subunits are widely distributed throughout the CNS, α6 subunit is found exclusively in the cerebellum[7,8]. The most common type of receptors that are located at synapses are composed of α1, β2, and γ2 subunits[9]. These synaptic GABA_A_Rs mediate the phasic inhibition, which is achieved through the presynaptic terminal release of GABA onto postsynaptic receptors that elicits a fast, transient inhibitory postsynaptic potential[10]. Conversely, GABA_A_Rs consisting of α4, α6, and δ subunits are generally located outside the synapse. These high-affinity, low-conductance extrasynaptic receptors are largely responsible for mediating the tonic inhibition, where ambient levels of GABA outside of synaptic sites generate a sustained, prolonged inhibitory current to regulate the overall neuronal excitability[11].

**Fig. 1.**
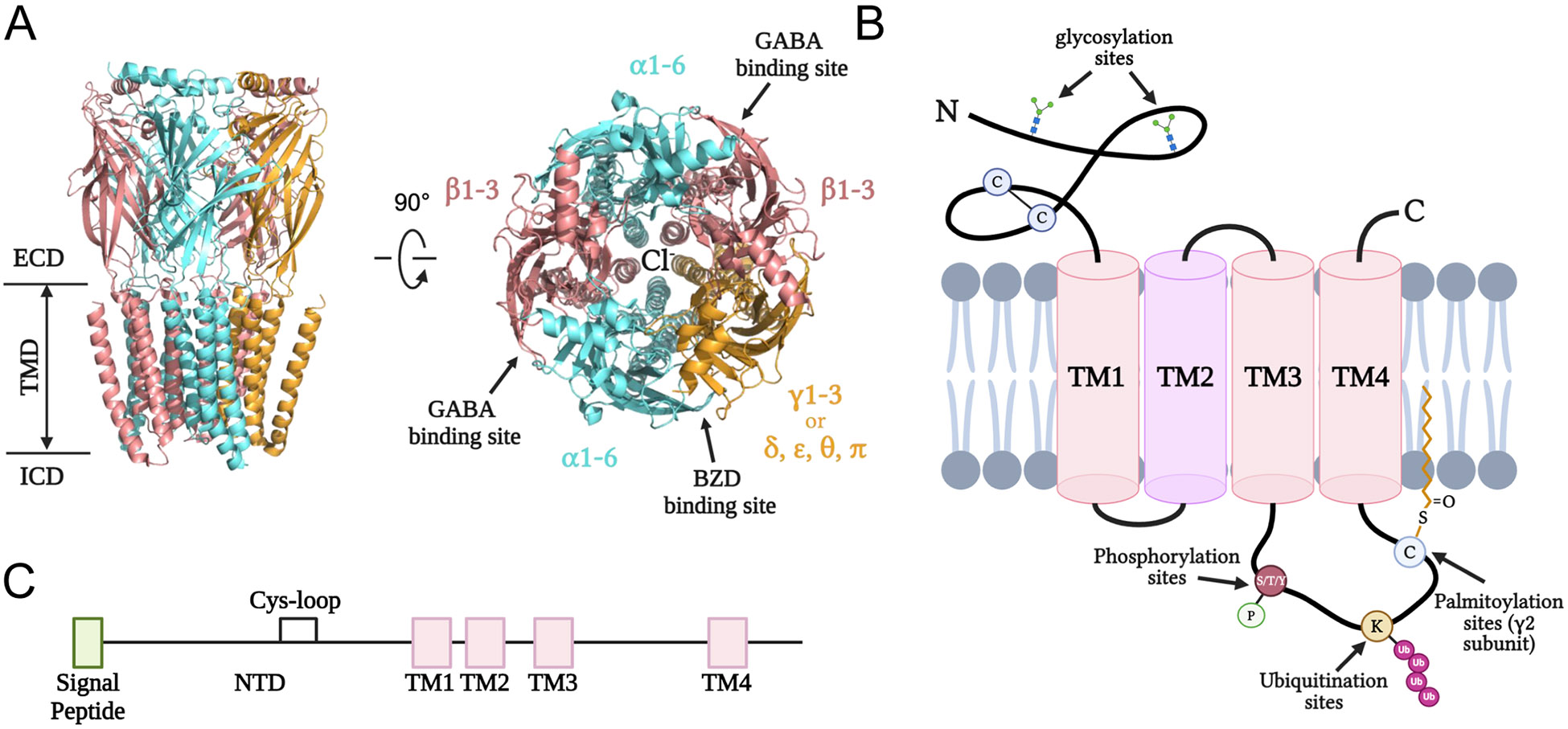
Structure of GABA_A_Rs. (A) Cartoon representation of the pentameric GABA_A_R, showing the large extracellular domain (ECD), transmembrane domain (TMD), and intracellular domain (ICD). GABA and benzodiazepine (BZD) binding sites are highlighted as viewed from the synaptic cleft on the right. PDB: 6X3S. (B) Topology of individual GABA_A_R subunit. Each subunit contains N-linked glycosylation sites and the signature Cys-loop in the N-terminal domain (NTD), four trans-membrane helices (TM1–4), and a large intracellular loop between TM3 and TM4 that is subjected to post-translational modifications including phosphorylation, ubiquitination, and palmitoylation. TM2 lines the pore of the receptor. (C) Linear representation of GABA_A_R subunit architecture.

Recent high-resolution cryo-electron microscopy (cryo-EM) studies solved the structures of the pentameric GABA_A_Rs including α1β2γ2 receptors[12] and α1β3γ2 receptors[13], which largely expanded our knowledge of receptor pharmacology and functionality. As viewed from the synaptic cleft, GABA_A_R subunits are arranged in a β-α-β-α-γ counterclockwise direction (Fig. 1A). Each subunit contains shared structural elements: a large extracellular N-terminal domain (NTD), four transmembrane helices (TM1–4), a short intracellular TM1–2 and extracellular TM2–3 loop, a long intracellular TM3–4 loop, and a short extracellular C terminus (Fig. 1B, C). NTD contains two α-helices, ten β-sheets (β1-β10), and many connecting loops. TM2 of each subunit lines the channel pore, and the large intracellular TM3–4 loop is a key site for many phosphorylation-dependent regulations and interaction with other proteins[14]. Two molecules of GABA bind at the β+ (principal) and α− (complementary) interfaces, which results in receptor activation, allowing the influx of chloride ions through the channel and membrane hyperpolarization. While the binding of one GABA molecule is sufficient to open the channel, when both α/β interfaces are occupied by GABA, the probability of channel opening increases dramatically[15].

## 2. Protein quality control of GABA_A_Rs

Proteostasis maintenance of GABA_A_Rs ensures their protein folding, assembly, trafficking, and degradation are balanced, and thus is essential for normal receptor activity in health[16]. Proteostasis deficiency of GABA_A_Rs, often caused by their disease-associated variants, leads to various neurological diseases[17]. Therefore, it is critical to decipher how the proteostasis network orchestrates the folding, assembly, trafficking, and degradation of GABA_A_Rs in health and disease. Here, we summarize known key players in the GABA_A_R proteostasis network that regulate the expression and function of GABA_A_Rs (Table 1). The knowledge of protein quality control of GABA_A_Rs is essential for designing mechanism-based therapeutic strategies to treat related diseases.

### 2.1. Folding and assembly of GABA_A_Rs

**Fig. 2.**
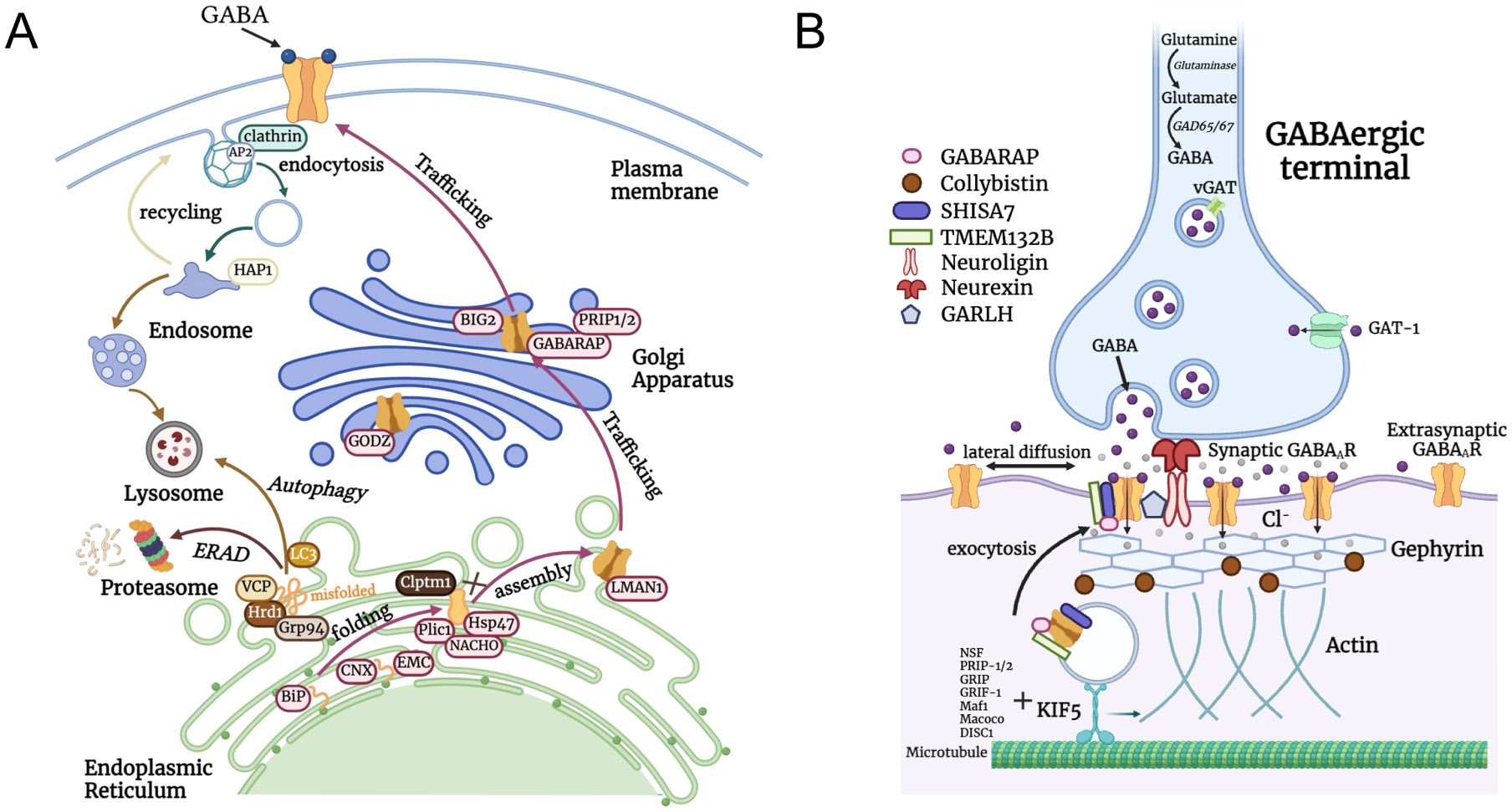
Proteostasis Regulation of GABA_A_Rs. (A) Newly synthesized GABA_A_R subunit is first folded into its proper structure with the aid of molecular chaperones and folding enzymes, such as BiP or calnexin (CNX). Correctly folded subunits are assembled as heteropentamers and further traffic through the Golgi apparatus to form functional receptors on the plasma membrane. Terminally misfolded subunits are targeted to proteasome- or lysosome-dependent degradation pathways. Surface receptors also undergo clathrin-dependent endocytosis. Internalized receptors are either transported through the late endosome to be degraded in the lysosome or recycled back to the plasma membrane. GABA_A_R-interacting proteins along each stage are highlighted. (B) Synaptic organization of GABA_A_Rs. GABA_A_Rs are transported in vesicles via microtubule-dependent motor kinesin KIF5. At the synapse, gephyrin plays a major role in anchoring the synaptic receptors, promoting synaptic strength and stability. Additionally, GABA_A_R auxiliary subunits, such as Shisa7, TMEM132B, and GARLH, contribute to synaptic clustering of GABA_A_Rs.

Upon the synthesis in the ribosome, each subunit must fold and assemble in the endoplasmic reticulum (ER) with the help of folding enzymes and assembly factors before anterograde trafficking to the Golgi and plasma membrane (Fig. 2A). Molecular chaperones play an indispensable role in assisting protein folding. Using an ATP-dependent mechanism, chaperones such as the Hsp70 system bind to exposed hydrophobic regions of unfolded or partially folded proteins to prevent protein aggregation and keep the protein in a folding-competent state. Binding immunoglobin protein (BiP/Grp78), a Hsp70 family protein in the ER, plays a major role in promoting protein folding and ER quality control. Previous studies found that BiP preferentially associated with the misfolding-prone α1(A322D) subunit, suggesting that BiP binds to unfolded proteins in the early biogenesis pathway to promote folding [18,19]. Additionally, overexpressing BiP promoted the forward trafficking and maturation of both WT and variant α1 subunit. Another mechanism of chaperone-mediated folding is lectin-dependent folding. Calnexin and calreticulin, which are both lectin proteins in the ER, recognize and bind to incompletely folded N-glycosylated proteins, retaining them for additional folding cycles within the ER[20]. GABA_A_R subunits have N-glycosylation sites with the sequon Asn-X-Ser/Thr, where X can be any residue except Pro, in the ER lumen (Fig. 1B). For example, α1 has two sites at Asn38 and Asn138, β2 has three sites at Asn32, Asn104, and Asn173, and γ2 has three sites at Asn52, Asn129, and Asn247. Calnexin and calreticulin overexpression enhanced the ER-to-Golgi trafficking efficiency of α1(D219N) subunit; moreover, the interaction between calnexin and α1(D219N) is dependent on the N-linked glycans since the glycosylation sites N38Q/N138Q double mutant in α1(D219N) diminished such an interaction[21]. A highly conserved region within the extracellular N terminus adjoining the first transmembrane domain of α1, β3, and γ2 subunit is shown to be crucial for receptor interaction with calnexin, thereby facilitating ER processing of GABA_A_Rs and anterograde trafficking to the cell surface[22]. Presumably, protein disulfide isomerases (PDIases) in the ER are essential for the formation of the signature Cys-loop of GABA_A_Rs; however, direct evidence showing the role of specific PDIases in the maturation of GABA_A_Rs in the ER is lacking. Additionally, GABA can serve as a ligand chaperone to promote receptor surface expression both in recombinant and endogenous systems[23,24]. Interestingly, both a GABA_A_R agonist (GABA) and a competitive antagonist (bicuculine) can behave as ligand chaperones to enhance receptor surface expression in HEK293T cells [23].

Once folded, each subunit needs to assemble with other subunits to form a heteropentamer before being delivered to the cell surface. Although β1 and β3 homopentameric channels could form, they are insensitive to GABA, as the α/β interface where GABA binds is critical for receptor function[25-28]. Additionally, expression of individual subunits (i.e., α1, β2, γ2 L) alone or only α1/γ2 L or β2/γ2 L leads to ER retention[29]. These findings suggest the importance of subunit assembly for proper receptor function. A recent study found that Hsp47 (gene name: *SERPINH1*), a heat shock protein residing in the ER lumen, binds after BiP to enhance the receptor assembly process[30]. Overexpression of Hsp47 enhanced the functional surface expression of endogenous GABA_A_Rs in primary neurons and epilepsy-associated GABA_A_R variants in HEK293T cells. Hsp47 preferentially interacts with the folded conformation of GABA_A_Rs, thus promoting the subunit-subunit interaction and ER-to-Golgi forward trafficking of GABA_A_Rs. In addition, the ER membrane protein complex (EMC), especially EMC3 and EMC6, facilitates the insertion of the TM helices of GABA_A_Rs into the lipid bilayer[31,32]. Presumably, the coordinated interactions of Hsp47 in the ER luminal region and EMC in the transmembrane domain ensure the efficient folding and assembly of GABA_A_Rs in the ER membrane. More recently, an ER-resident membrane protein NACHO (gene name: *TMEM35A*) was also found to be essential for the stepwise assembly of pentameric ligand-gated ion channels like GABA_A_Rs[33], although NACHO was initially identified as a specific chaperone for nAChRs[34]. It was proposed that NACHO first interacts with the plus side of the folded α1 subunit and homodimerizes with another α1-NACHO complex. The NACHO-α-α-NACHO complex then engages and recruits the β2 subunits. The interaction between the extracellular domains (ECDs) would then favor the displacement of NACHO, allowing the association of the final subunit, γ2. Nonetheless, many of the fundamental aspects of how the assembly chaperones regulate the assembly of GABA_A_Rs remain to be established.

### 2.2. ER-associated degradation (ERAD) and autophagy of GABA_A_Rs

The correctly assembled heteropentamers further traffic through the Golgi apparatus to the plasma membrane to form a functional receptor. Misfolded or unassembled subunits remain in the ER for additional folding/assembling cycles, and terminally misfolded or unassembled subunits will be removed from the ER via cellular degradation pathways (Fig. 2A). ER-associated degradation (ERAD) pathway is one such pathway to target misfolded or unassembled GABA_A_R subunits to the ubiquitin-proteasome system (UPS) for degradation[35-37]. ERAD involves the recognition of unfolded proteins in the ER, retro-translocation into the cytosol, covalent conjugation with polyubiquitin, and proteasomal degradation. Various proteins play an important role in this, including ER luminal, ER membrane-associated and cytosolic chaperone proteins that recognize misfolded proteins and E3 ubiquitin ligases that conjugate ubiquitin on the targeted substrates. Previous studies found that glucose-related protein 94 (Grp94), a Hsp90 in the ER lumen, interacts with the misfolded α1(A322D) subunit of GABA_A_Rs to deliver it to the Hrd1-mediated ubiquitination pathway[35]. OS-9, an ER resident lectin, also recognizes misfolded α1 subunit in a glycan-dependent manner to promote ERAD. Knockdown of E3 ligase Hrd1/SYVN1 decreased ubiquitination levels and increased the total α1 protein level, indicating that Hrd1 plays a major role in the degradation of α1 subunit [35,38]. Additionally, knocking down TRIM21, an E3 ubiquitin ligase, selectively attenuated the ERAD of α1(A322D) subunit, but not WT α1 subunit[16]. Ring finger protein 34 (RNF34), an E3 ubiquitin ligase, specifically interacts with γ2 subunit to promote ubiquitination and subsequent degradation of GABA_A_Rs[39]. Another E3 ubiquitin ligase TRIM9 was shown to regulate synaptic receptor levels, as loss of TRIM9 increased surface and total protein expression of GABA_A_R α1 subunit [40]. The ubiquitination sites of GABA_A_Rs presumably reside in their large TM3–4 intracellular loops (Fig. 1B); however, the critical Lys residues for ubiquitination remain to be determined. Furthermore, the AAA + ATPase valosin-containing protein (VCP)/p97 retrograde translocates misfolded proteins from the ER to the cytosol, thus subjecting misfolded α1 subunits to degradation[41]. In contrast, the ubiquitin-like protein Plic-1 was found as a negative regulator of ERAD. It directly interacts with GABA_A_Rs to inhibit ubiquitination, thereby positively regulating membrane trafficking of GABA_A_Rs and promoting membrane insertion[42,43].

In addition to the UPS degradation pathway, aggregation-prone GABA_A_Rs could be targeted to the lysosome for degradation via autophagy, ER-phagy, or ER-to-lysosome associated degradation[44-47]. Large protein aggregates that are too bulky to fit in the proteasome are preferentially degraded by autophagy in the lysosome[48,49]. ER-phagy, a selective form of autophagy that targets the specific components of the ER for degradation, is crucial for preserving ER homeostasis and ensuring protein quality control[50,51]. When ERAD is overwhelmed or impaired, ER-phagy may act as a compensatory mechanism to remove unwanted cargos[52]. It is mediated mainly by ER-phagy receptors, which interact with the autophagy-related protein 8 (ATG8) family proteins, including LC3 and GABARAP, through the LC3-interacting region (LIR). Damaged proteins are encapsulated into autophagosomes that fuse with lysosomes to form autophagic lysosomes for degradation by acid hydrolases. However, specific factors that are involved in autophagy of GABA_A_Rs remain to be elucidated.

Recent studies identified Cleft lip and palate transmembrane protein 1 (Clptm1) as a negative regulator of the forward trafficking of GABA_A_Rs by trapping the receptors in the ER. Clptm1 overexpression decreased GABA_A_R-mediated currents without affecting glycine or AMPA receptor-mediated neurotransmission, while knocking down Clptm1 enhanced phasic and tonic inhibitory currents [53]. Subsequent in vivo studies found that Clptm1 knock-out (KO) mice had elevated phasic and tonic inhibitory transmission and associated cognitive deficits[54], and the downregulation of Clptm1 expression protected against pentylenetetrazol-induced epilepsy in rat[55]. However, de novo Clptm1 variants are associated with epilepsy and shown to reduce surface expression of the GABA_A_R γ2 subunit and GABA_A_R current response under voltage clamp[56]. Nonetheless, whether Clptm1 directly interacts with GABA_A_Rs and the molecular mechanism of Clptm1 in the degradation or trafficking of GABA_A_Rs merits further investigation.

### 2.3. GABA_A_R trafficking and regulation by post-translational modifications

After assembling with other subunits, the heteropentameric receptors further traffic from the ER to the Golgi apparatus. In the Golgi, the receptors interact with many protein factors and undergo additional post-translational modifications before being transported to the cell surface (Fig. 2A). An important post-translational modification that occurs in the Golgi is palmitoylation, which attaches palmitate to Cys through a thioester bond (Fig. 1B). The γ2 subunit of GABA_A_Rs undergoes palmitoylation on multiple cysteine residues by the GODZ (Golgi-specific DHHC zinc finger protein, ZDHHC3) and its close paralog sertoli cell gene with a zinc finger domain-β (SERZ-β, ZDHHC7). GODZ-mediated palmitoylation of GABA_A_Rs is required for correct assembly and synaptic GABAergic inhibitory function[57,58]. Overexpression of a dominant negative GODZ or depleting GODZ with siRNA disrupts the postsynaptic accumulation of GABA_A_Rs[57]. GODZ KO mice also demonstrated a decreased accumulation of GABA_A_Rs at synapses, GABAergic innervation, and synaptic function[59].

Post-Golgi GABA_A_Rs are further packaged into vesicles for transport to and insertion into the plasma membrane (Fig. 2A). GABA_A_-receptor-associated protein (GABARAP), which specifically interacts with the γ2 subunit of GABA_A_R, plays a significant role in promoting GABA_A_R surface trafficking and clustering of the receptor at synapses[60,61]. Overexpression of GABARAP increased the surface levels of GABA_A_Rs in neurons by enhancing their intracellular transport[62]. GABARAP also associates with microtubules and *N*-ethylmaleimide-sensitive factor (NSF), a protein that is involved in intracellular membrane fusion. NSF has been shown to directly bind to the β subunit of GABA_A_R and may act with GABARAP to collectively enhance GABA_A_R surface trafficking[63]. Additionally, phosphorylation of the γ2 subunit regulates the receptor binding to GABARAP and the clathrin adaptor protein AP2, thus promoting receptor forward trafficking and modulating synaptic localization of the receptor[64].

BIG1, brefeldin A-inhibited guanine nucleotide-exchange factor 1, plays an important role in GABA_A_R trafficking. Loss of BIG1 using siRNA reduced GABA_A_R expression at the neuronal surface and impaired GABA-gated chloride influx[65]. Additionally, BIG2 specifically interacts with the intracellular loop of GABA_A_R β subunit as shown by a yeast two-hybrid assay[66]. BIG2 is located in the trans-Golgi network and promotes the trafficking of assembled GABA_A_Rs from the Golgi to the cell surface.

Phospholipase C-related catalytically inactive proteins (PRIP1 and PRIP2) are inositol 1,4,5-trisphosphate binding proteins that were shown to associate with GABARAP to promote the transport of γ2-containing GABA_A_Rs to the surface[67]. PRIP-1 and PRIP-2 double knock-out (PRIP-DKO) mice displayed reduced interaction between GABARAP and GABA_A_R and decreased diazepam sensitivity. In addition, PRIPs can modulate the function of GABA_A_Rs by regulating their phosphorylation. PRIP1 inactivates protein phosphatase 1α (PP1α), which dephosphorylates GABA_A_Rs to terminate phosphorylation-dependent receptor regulation[68]. PRIP-1 KO mice were shown to have impaired protein kinase A (PKA)-dependent potentiation of GABA_A_R--mediated current.

In addition to phosphorylation-dependent modulation by PRIPs, GABA_A_Rs are subjected to dynamic regulations by various kinases and phosphatases that alter the surface expression and synaptic activity of the receptor. The long intracellular TM3–4 loop contains numerous consensus sites for phosphorylation by both serine/threonine and tyrosine protein kinases (Fig. 1B). For example, a conserved serine residue in β1–3 subunits can be phosphorylated by PKA, protein kinase C (PKC), protein kinase G (PKG), and Ca^2+^/calmodulin-dependent protein kinase II (CaMKII)[69], which can affect receptor surface expression and functional activity. PKA phosphorylates β3(S408/S409) subunits to potentiate GABA_A_R-mediated responses, while phosphorylation of β1 (S409) seems to have the opposite effect[70]. Phosphorylation of GABA_A_R β3(S383) subunit promoted the insertion of GABA_A_R on the surface and increased tonic current[71]. On the other hand, calcineurin dephosphorylates γ2 subunits of GABA_A_R to induce long-term depression (LTD) at inhibitory synapses and mediate the reduced surface level of GABA_A_Rs[72,73]. In vivo model showed that kainic acid-induced status epilepticus results in elevated calcineurin activity, which downregulates GABA_A_R activation to promote seizures in rats [74].

Intracellular trafficking often involves the help of motor proteins, which are essential for moving cellular cargoes, including GABA_A_Rs within the cell (Fig. 2B). GABA_A_R-interacting factor 1 (GRIF-1) belongs to the TRAK family of proteins that interact with kinesin and dynein-dynactin motor protein complexes involved in intracellular trafficking [75]. GRIF-1 (also known as TRAK2) specifically interacts with GABA_A_R β2 subunit[76]. In addition, TRAK1 protein is shown to regulate motor-dependent transport of GABA_A_Rs, as TRAK1 mutant mice display hypertonia and loss of GABA_A_R expression in the brain and motor neurons[77]. Additionally, the microtubule-dependent motor kinesin-related protein 5 (KIF5) family proteins, including KIF5A, KIF5B, and KIF5C, play a significant role in the vesicular transport of GABA_A_R to the synapses. The adaptor protein HAP1 (huntingtin-associated protein 1) links the receptor to KIF5, and the disruption of the HAP1-KIF5 complex led to a decrease in synaptic GABA_A_R number[78]. Loss of KIF5A in mice resulted in reduced neuronal GABA_A_R surface expressions, impaired GABA_A_R-mediated synaptic transmission, and associated epileptic phenotypes[79].

### 2.4. GABA_A_R clustering at synapses

The clustering of GABA_A_Rs at synapses ensures a high density of receptors at the post-synaptic sites and is critical for efficient synaptic inhibition and maintenance of excitation-inhibition balance (Fig. 2B). This process is largely mediated by the scaffolding protein gephyrin. Gephyrin is widely expressed in neuronal tissues and enriched in postsynaptic inhibitory GABAergic and glycinergic synapses[80]. Gephyrin was first identified to be associated with β subunit of GlyRs[81], but later was also found to bind to GABA_A_Rs at inhibitory synapses[81,82]. Previous studies found that gephyrin-deficient mice demonstrated loss of GABA_A_R clusters containing α2 or γ2 subunits at the synaptic sites, albeit with minimal change in functional GABA_A_R expression[83]. Additionally, key residues in the TM3–4 loops of α1, α2, and α3 subunits were identified that mediate the interaction between GABA_A_R and gephyrin. Such residues appear to be the major site responsible for gephyrin-dependent clustering of GABA_A_R, although other subunits may also be involved[82,84,85]. Collybistin is a known binding partner for gephyrin, and the loss of collybistin in mice led to reduced GABA_A_R clustering, defective synaptic plasticity, and impaired GABAergic transmission[86]. These observations suggest that gephyrin anchors and stabilizes α2/γ2-containing GABA_A_R clusters at synapses. Additionally, during inhibitory postsynaptic long-term potentiation (LTP), gephyrin accumulates at synaptic sites[87], while inhibitory synaptic depression is linked to the dispersion of gephyrin and lateral diffusion of GABA_A_Rs [88], indicating the role of gephyrin in regulating synaptic plasticity.

Although gephyrin is important in promoting synaptic GABA_A_R clustering, previous studies also reported the existence of clustered receptors and miniature inhibitory postsynaptic currents (mIPSCs) in gephyrin −/− neurons[89], suggesting gephyrin-independent clustering mechanisms. GABA_A_Rs containing α5 subunit accumulate at the extrasynaptic sites via radixin-mediated anchorage at the actin skeleton[90]. Radixin-mediated clustering involves membrane association and a phosphorylation-dependent conformational change from RhoA/ROCK signaling. Several studies also suggest that calcineurin regulates lateral mobility of GABA_A_R by dephosphorylating S327 on γ2 subunit of GABA_A_R, thereby controlling the number of synaptic GABA_A_Rs independent of gephyrin[91,92]. Additionally, neuroligin 2, a synaptic-specific adhesion molecule, selectively localizes to inhibitory synapses and associates with GABA_A_Rs[93]. It is also a part of the intracellular scaffolds that regulate postsynaptic GABA_A_R accumulation and inhibitory neurotransmission. Neurexins, which are presynaptic cell-adhesion molecules, form complexes with neuroligins that are on the postsynaptic membrane. They interact with postsynaptic GABA_A_Rs and suppress GABA_A_R-mediated synaptic transmission[94].

Other proteins that are critical for GABA_A_R synaptic localizations are GARLH (GABA receptor-like homolog) and Neurobeachin proteins. GARLH family consists of GARLH3 and GARLH4 proteins. GARLH4 bridges γ2-containing GABA_A_Rs and neuroligin-2 in the brain and is critical for the synaptic localization of GABA_A_Rs and inhibitory synaptic transmission[95,96]. Neurobeachin, a brain-specific A-kinase anchor protein (AKAP), also modulates synaptic surface expression of GABA_A_Rs, although the specific mechanism is not yet clear[97].

Additionally, Shisa7, a single-pass transmembrane protein, was identified as an auxiliary subunit for GABA_A_Rs that regulates GABA_A_R trafficking and neurotransmission. It localizes to inhibitory synapses and associates with GABA_A_R through a distinctive N-terminal domain[98]. Shisa7 also interacts with α5 subunit of GABA_A_Rs and regulates tonic inhibition[99]. Importantly, the phosphorylation of Shisa7 at S405 plays a crucial role in GABAergic transmission and plasticity since phospho-deficient mice demonstrated reduced α1/α2/α5-containing GABA_A_R surface expression[100]. Consequently, the altered GABA_A_R activity in phospho-deficient mice was linked to behavioral phenotypes as seen in neurodevelopmental disorders, such as hyperactivity and impaired sleep homeostasis. Shisa7 also potentiates the action of diazepam, and the loss of Shisa7 abolishes the effect of diazepam in GABAergic transmission in vivo and in vitro[98]. Single-channel kinetics analysis revealed that Shisa7 modulates GABA_A_R channel gating by accelerating GABA_A_R deactivation and reducing the frequency, duration, and open probability of the channel[101].

Another GABA_A_R auxiliary subunit is TMEM132B[102]. TMEM132B, a single-pass transmembrane protein, interacts with various GABA_A_R subtypes in heterologous cells, localizes at GABAergic synapses, and promotes cell surface expression of GABA_A_Rs. TMEM132B deficiency in mice hippocampal neurons diminished GABAergic transmission and further abrogated alcohol-induced potentiation of GABA_A_R-mediated currents.

### 2.5. GABA_A_R endocytosis

Endocytosis or internalization of cell surface receptors modulates surface receptor levels and controls signaling duration and intensity. The endocytosed receptors reach early endosomes and then are either recycled back to the plasma membrane or targeted to the lysosome for degradation via the late endosomes[103,104]. Clathrin-mediated endocytosis is the main internalization mechanism for GABA_A_Rs, which is largely dependent on the clathrin adaptor protein AP2 (Fig. 2A). AP2 interacts with the intracellular loops of β and γ subunits of GABA_A_Rs[105]. Previous studies reported that AP2 binds to a dileucine motif within the β2 subunit and a motif containing three arginine residues (^405^RRR^407^) within the β3 subunit intracellular domain to regulate GABA_A_R internalization[106,107]. The β3 binding site contains residues (S408/S409) that can be phosphorylated by PKA and PKC, and the phosphorylation of these residues drastically reduces the binding affinity to AP2[108]. Disruption of the AP2 binding domain leads to reduced endocytosis and increased surface levels of the receptor. Additionally, tyrosines 365/367 in the GABA_A_R γ2 subunit was found to be an AP2 binding site[109], which is also subjected to phosphorylation by Src kinases. Phosphorylation of this site weakens the interaction between AP2 and γ2 subunit, which consequently increases synaptic receptor levels and mIPSC amplitude[109]. Overall, these findings demonstrate that the phosphorylation of GABA_A_R subunits positively modulates the availability of GABA_A_R β2, β3, and γ2 subunits on the surface and the efficacy of synaptic inhibition.

After endocytosis, the recycling of GABA_A_Rs back to the plasma membrane helps maintain proper receptor levels at synapses and modulate inhibitory synaptic strength. HAP1, a cytoplasmic protein with several central coil-coiled domains, interacts with the intracellular loop of GABA_A_R β subunits to inhibit receptor degradation and promote receptor recycling[110]. Overexpression of HAP1 reduced GABA_A_R degradation and increased surface receptor level[110]. Additionally, giant ankyrin-G (ANK3) interacts with GABARAP and stabilizes GABA_A_Rs at somatodendritic synapses by inhibiting their endocytosis [111].

### 2.6. Lipids involvement in GABA_A_R trafficking and function

Lipids play an important role in modulating GABA_A_R dynamics and function. Membrane cholesterol levels can impact GABA_A_R functionality, as both cholesterol enrichment and depletion reduced the potency of GABA[112]. In resting state, GABA_A_R associates with lipid rafts, and the palmitoylation of γ subunit by GODZ promotes GABA_A_R presence within the lipid rafts[113]. Super-resolution imaging showed that GABA induces GABA_A_R translocation from lipid rafts to phosphatidylinositol 4, 5-bisphosphate (PIP2) clusters in mouse primary cortical neurons[113]. Indeed, cryo-EM structures of human α1β3γ2 GABA_A_ receptor showed that the pentameric receptor is bound to two PIP2 molecules, which seems to modulate receptor trafficking, rather than affecting the channel function[13]. Phospholipids (i.e., phosphatidylserine[114]) and polyunsaturated fatty acids[115] also affect GABA_A_R activity and stability by regulating the lipid bilayer elasticity. Changes in lipid bilayer elasticity can affect GABA_A_R binding ability and rate of receptor desensitization. Additionally, certain neurosteroids, such as allopregnanolone, bind to GABA_A_R at distinct binding sites (β(+)–α(−) interface) and serve as positive allosteric modulators to enhance GABA-mediated chloride currents[116]. Moreover, neurosteroids can enhance the PKC-dependent phosphorylation of S443 within α4 subunits to potentiate trafficking of GABA_A_Rs responsible for tonic inhibition[117]. Conversely, other neurosteroids (i.e., 3β-OH pregnane steroids) bind at intrasubunit sites and serve as GABA_A_R antagonists to inhibit receptor activation. Thus, neurosteroids can modulate GABA_A_ receptor trafficking and function in a site-specific manner[116].

## 3. GABA_A_Rs as a drug target

**Fig. 3.**
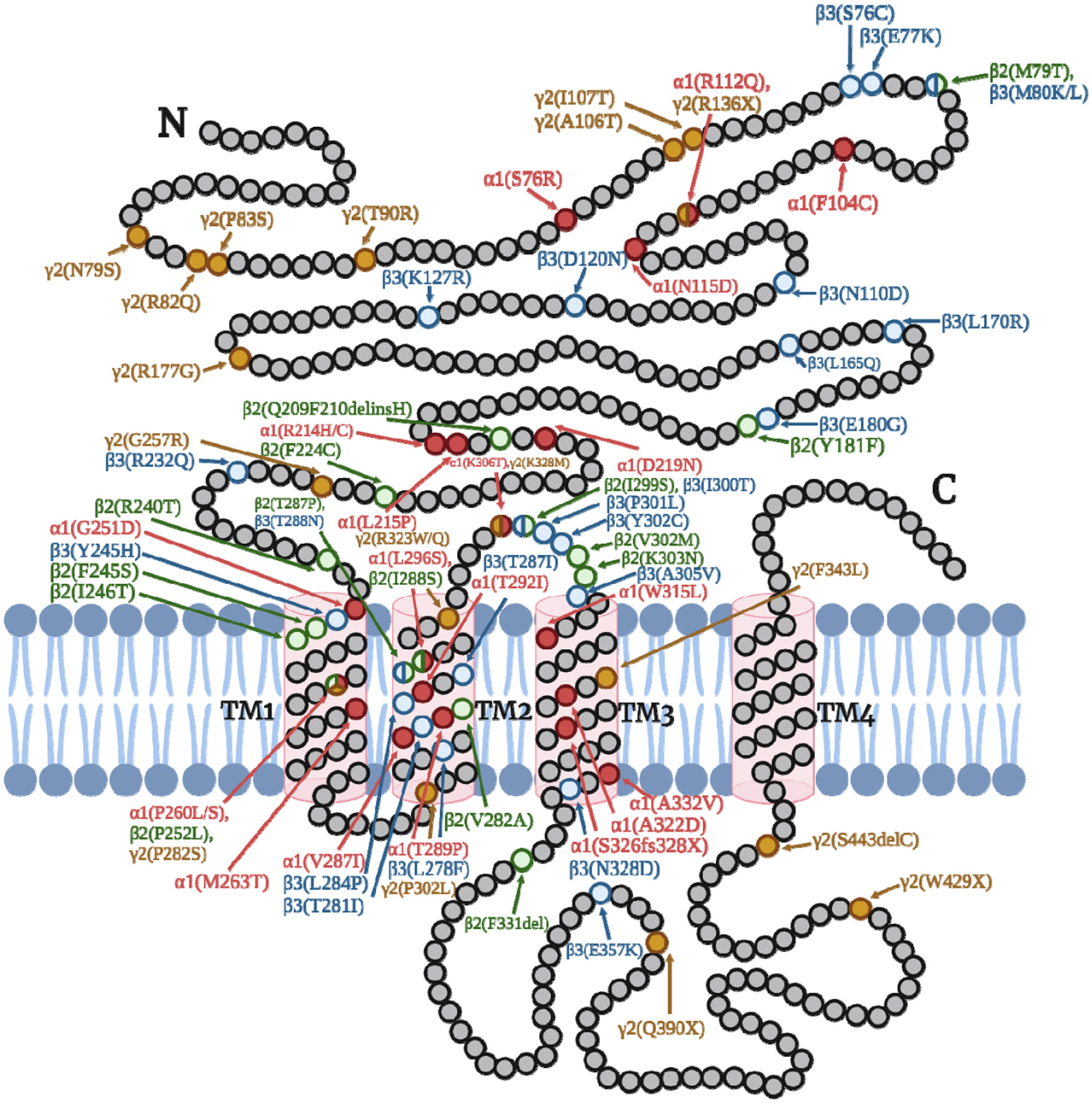
Schematic representation of the subunit topology of a GABA_A_R subunit, showing the locations of currently known epilepsy-associated variants in major GABA_A_R subunits (α1, β2/3, and γ2). Variants in α1, β2, β3, and γ2 subunits are highlighted in red, green, blue, and yellow, respectively. A few homologous residues have reported variations in multiple subunits, as indicated by the color overlap. Variant locations in all subunits are numbered starting from the beginning of the precursor protein, which includes the signal peptide.

Because GABA_A_Rs play a critical role in neurotransmission, defects in GABA_A_R-mediated inhibition can lead to a wide range of neurodevelopmental and neuropsychiatric disorders, including autism[118, 119], epilepsy[17,120], anxiety disorders[121], depression[122,123], schizophrenia[124,125], and bipolar disorder[126]. Importantly, over 1000 clinical variations in genes encoding GABA_A_R subunit have been reported in ClinVar[127], which contribute to various neurological disorders ranging from mild febrile seizures to severe epileptic encephalopathy[128,129]. Wang et al. summarized the pathogenicity of disease-associated missense variants in genes encoding *GABRA1*, *GABRB2*, *GABRB3*, and *GABRG2*, using state-of-the-art computational tools[129]. In addition, variants in other GABA_A_R subunits, such as *GABRA2–6*, *GABRB1*, and *GABRD*, have been identified to be associated with epilepsy[130-135]. GABA_A_R variants can influence their molecular functions, including mRNA stability, proteostasis processes (protein folding, degradation, aggregation, stability, subunit assembly, and anterograde trafficking to the surface), and electrophysiological properties (peak current amplitudes, ligand potency, and current kinetics). The known molecular functional deficiencies of pathogenic GABA_A_R variants in α1, β2, β3, and γ2 subunits were summarized in Table 2, and their spatial distribution in the primary protein sequences was visualized in Fig. 3. In total, 24 α1 variants, 15 β2 variants, 23 β3 variants, and 19 γ2 variants were listed in Table 2. For example, α1(D219N) and α1 (A322D) variants are associated with familial juvenile myoclonic epilepsy (JME). These variants impair the folding and assembly of the subunits, resulting in reduced α1 surface expression and rapid degradation[19,21,136]. Similarly, γ2(R177G) and γ2(R82Q) variants, which are associated with childhood absence epilepsy (CAE), demonstrate impaired subunit folding and subunit assembly process, leading to selective ER retention[137,138]. CAE-linked β3 heterozygous missense variations (P11S, S15F, G32R) showed hyperglycosylation patterns and reduced GABA-evoked current, possibly due to an altered maturation and trafficking of GABA_A_R from ER to the cell surface[139]. Many pathogenic *GABRB3* gain-of-function or loss-of-function missense variants have since been identified, which are associated with distinct clinical phenotypes[140]. However, the definition of gain-of-function variants in GABA_A_Rs needs to be clarified by considering proteostasis defects and multiple electrophysiological properties. Additionally, nonsense variants, frameshift variants, and certain intronic variants can generate a pre-mature stop codon, which triggers nonsense-mediated mRNA decay (NMD), such as in the case of the CAE-associated intronic *GABRG2* variant, IVS6 +2T->G[141]. The truncated proteins translated from variant mRNA that was able to escape NMD are often misfolded, trafficking deficient, and efficiently removed via degradation pathways. Therefore, GABA_A_R is one of the most critical drug targets in the treatment of these devastating neurological and neuro-developmental diseases. Correcting the folding, assembly, and trafficking of these misfolding-prone receptors represents a novel therapeutic approach to ameliorate many neurodevelopmental diseases associated with pathogenic GABA_A_Rs.

### 3.1. Allosteric modulators and pharmacological chaperones of GABA_A_Rs

Numerous ligands aside from GABA have been shown to bind to diverse locations within each subunit to positively or negatively regulate receptor function[8]. Allosteric modulators of GABA_A_R bind to allosteric sites that are distinct from GABA-binding pockets. GABA_A_R positive allosteric modulators (PAMs) enhance the inhibitory effects of the receptor by increasing the frequency and/or the duration of chloride channel opening when an agonist is bound [142]. This leads to enhanced hyperpolarization in the postsynaptic neuron, which decreases excitability and likelihood of action potential firing. PAMs, such as benzodiazepines, barbiturates, ethanol, non-benzodiazepine hypnotics, and induction anesthetics, are used widely clinically to produce sedation, anticonvulsant, anxiolytic, and muscle relaxant effects and target various GABA_A_R-related disorders including seizures, anxiety, schizophrenia, and alcohol withdrawal. On the contrary, negative allosteric modulators (NAMs) of GABA_A_Rs block channel activity, which results in convulsions, neurotoxicity, and anxiety. These NAMs include picrotoxin, bicuculline, flumazenil, and MRK-016, which is selective for α5-containing GABA_A_Rs[143]. Endogenous neurosteroids can also serve as either PAMs or NAMs as described previously. Additionally, a recent study identified netrin-1 as an endogenous allosteric modulator of GABA_A_Rs [144]. Specifically, netrin-1 is secreted in response to elevated neuronal excitability and binds to the extracellular domains of GABA_A_R, enhancing receptor single-channel conductance without altering its surface expression. This demonstrates that, in addition to the numerous modulatory sites in the intracellular loop of the subunit, GABA_A_Rs can also be modulated through extracellular protein-protein interactions.

**Fig. 4.**
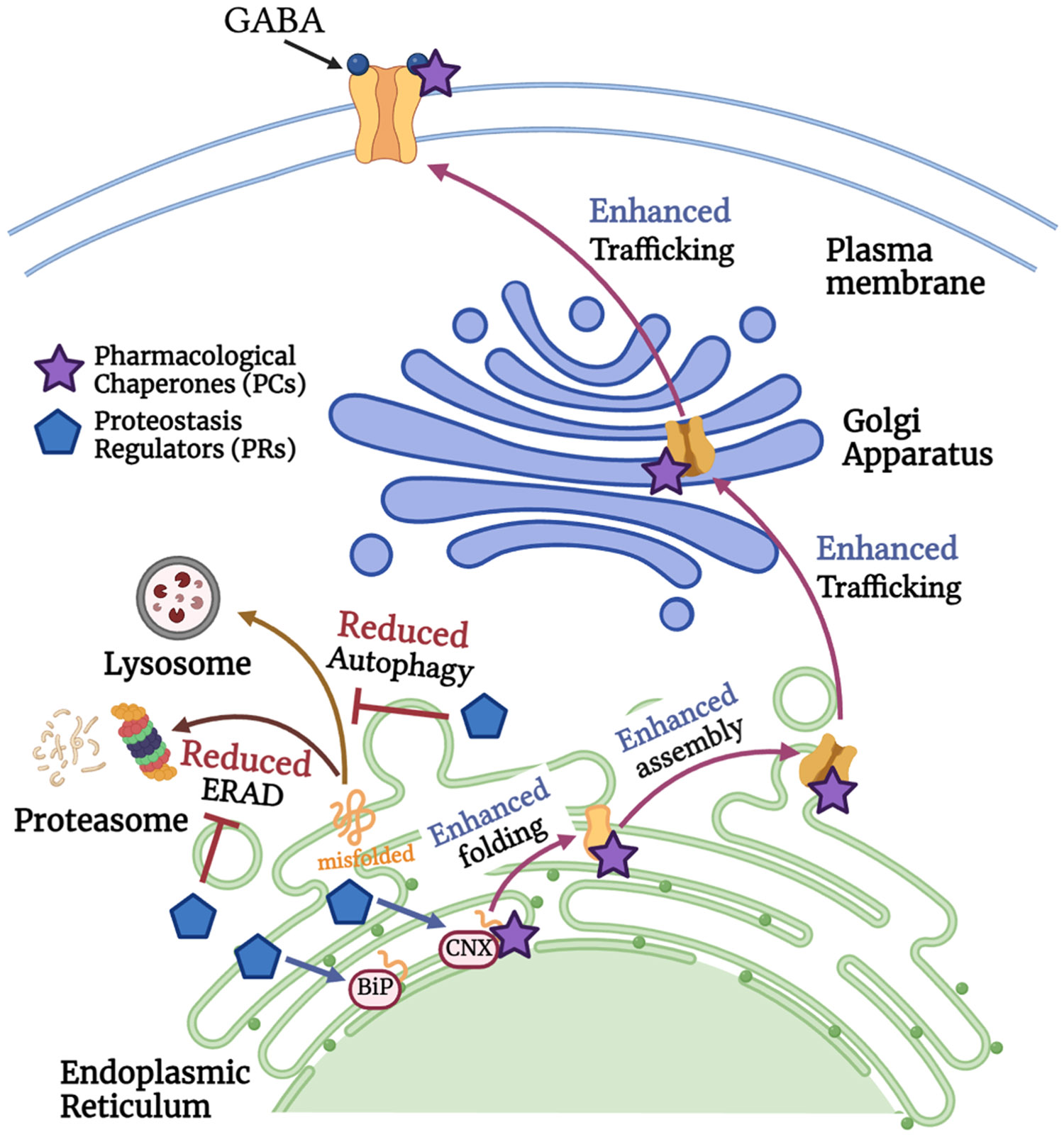
Current therapeutic approach for targeting epilepsy-associated GABA_A_R variants: pharmacological chaperones and proteostasis regulators. Pharmacological chaperones (PCs, shown in purple) are small molecules that interact with GABA_A_R protein in the early biogenesis pathway. Alternatively, proteostasis regulators (PRs, shown in blue) act as general protein folding enhancers to upregulate the expression of molecular chaperones. Despite their distinct mechanisms of action, both PCs and PRs enhance the folding, assembly, and trafficking of misfolding-prone GABA_A_R variants, while reducing their degradation. These molecules hold significant potential to target epilepsy-associated GABA_A_R variants to ameliorate channelopathies.

Pharmacological chaperones (PCs) are small molecules that bind directly to client proteins to stabilize them and enhance proper folding and trafficking[145] (Fig. 4). PCs have been developed as a treatment for various diseases such as cystic fibrosis, phenylketonuria, and lysosomal storage disorders, including Fabry disease, Gaucher disease, and Pompe disease[146]. Furthermore, GABA_A_R-specific PCs (i.e., hispidulin and TP003) have been demonstrated to correct the folding, assembly, and trafficking of various misfolding-prone α1 variants to greatly promote their functional surface expression[147]. These small molecules have the capacity to enhance the binding of α1 variant to pro-folding chaperones (i.e., BiP), while reducing α1 variant interactions with degradation factors (i.e., Grp94, VCP). Blood-brain-barrier-permeable PCs hold great therapeutic potential to treat genetic epilepsy caused by GABA_A_R variants in a receptor-specific manner.

### 3.2. Proteostasis regulators of GABA_A_Rs

Different from PCs, proteostasis regulators (PRs) remodel the cellular proteostasis network to correct protein folding, assembly, and trafficking defects, often by upregulating chaperone proteins[148,149] (Fig. 4). Examples of PRs for GABA_A_R variant-related channelopathies include HDAC inhibitors (e.g., SAHA)[18,150], calcium channel blockers (e.g., verapamil)[21], BiP activators (e.g., BIX) [151], and certain FDA-approved drugs (e.g., dinoprost, dihydroergocristine, and 4-phenylbutyric acid (4-PBA)) [152,153]. Additionally, PRs can adapt the intrinsic cellular stress response pathways to promote the folding, assembly, and trafficking of GABA_A_Rs, such as by activating the unfolded protein response (UPR)[154-156]. UPR consists of three signaling arms that are mediated by ER membrane proteins: IRE1 (Inositol-Requiring Enzyme 1), ATF6 (Activating Transcription Factor 6), and PERK (Protein Kinase R-like ER Kinase). UPR aims to adapt ER stress and restore protein homeostasis by reducing protein synthesis, improving protein folding capacity, and increasing degradation of misfolded proteins via the ERAD machinery. Previous studies found that pharmacologically activating the ATF6 branch using AA147 and AA263 promotes the folding, trafficking, and function of several trafficking-deficient α1 and γ2 variants[157]. These small molecules do not directly interact with GABA_A_Rs but instead modify the cellular environment to enhance proper folding and trafficking of the variant-containing receptors.

Since PRs and PCs have different mechanisms of action, co-application of PRs and PCs can synergistically or additively enhance the folding and trafficking of variant proteins. Mu et al. found that PCs for lysosomal enzyme glucocerebrosidase (GC) (i.e., N-(n-nonyl)deoxynojirimycin) stabilized the folded state ensemble of GC variants in the ER to increase its folding and trafficking capacity[158]. On the other hand, GC PRs (i.e., MG132 and celastrol) activated UPR to support the folding and trafficking of GC variants. Further, combining these PRs and PCs exhibited synergy in rescuing GC variant function, since PR upregulation of UPR promoted a larger pool of folded variant that PC can bind to and stabilize the trafficking-competent GC. However, combining PCs and PRs has not been reported for GABA_A_R variants. Therefore, it would be highly valuable to co-apply PCs and PRs of GABA_A_R to restore the activity of epilepsy-associated variants. By rescuing the function of variant receptors, these compounds can collectively repair the disrupted inhibitory neurotransmission.

### 3.3. Disease models for GABA_A_R variants

Human induced pluripotent stem cells (iPSCs) can be differentiated into cortical neurons, making them a valuable cellular system for modeling brain disorders and conducting drug screening studies[159]. Since human iPSCs can be derived from patients with neurological diseases and closely mimic the neuronal environment, iPSCs allow us to better characterize and study GABA_A_R-related disorders in an endogenous environment, compared to previous studies using HEK293T cells and other heterologous systems. For example, Wang et al. induced isogenic human iPSCs carrying the heterozygous GABA_A_R α1(G251D) variant into a highly pure population of GABAergic neurons by expression of transcription factors (i.e., ASCL1 and DLX2). They found that small molecule PCs (i.e., hispidulin and TP003) enhanced the forward trafficking of these epilepsy-associated human iPSC-derived GABAergic neurons[147]. Similarly, pharmacologically activating the ATF6 pathway of the UPR by AA147 and AA263 significantly increased the surface expression of human iPSC-derived cortical neurons expressing GABA_A_R γ2(R82Q) variant[157]. Kamand et al. reported the generation of patient-derived iPSCs carrying GABA_A_R δ(T291I) variant, as well as the isogenic control using CRISPR-Cas9 technique[160]. The development of human iPSC disease models for GABA_A_R variants is emerging, and there is currently only a limited number of studies on human iPSCs harboring disease-associated GABA_A_R variants and their neuronal differentiation. Given the critical role of GABA_A_Rs in neuronal inhibition and epilepsy pathology, there is an urgent need for further studies using human iPSCs carrying disease-associated GABA_A_R variants. Expanding research on human iPSCs with these variants will not only enrich our understanding of the molecular mechanisms underlying epilepsy, but also facilitate the development of more targeted and effective therapeutic strategies. These research efforts will enable us to identify personalized treatment plans for patients with specific genetic variants.

Additionally, the development of mouse models is important to enhance our understanding of GABA_A_R-related diseases on a wholeorganism level. As an example, studies on heterozygous α1(A322D) knock-in mice found decreased α1 subunit expression and reduced mIPSC peak amplitudes[161]. These mice experienced absence seizures, which closely mimic JME seen in patients with this variant. Similarly, *Gabrb3*^+*/N110D*^ knock-in mice displayed reduced cortical mIPSCs and epileptic spasms, seizures, and other neurological impairments consistent with infantile spasms syndrome[162]. Heterozygous *Gabrb3*^+*/D120N*^ knock-in mice had spontaneous atypical absence seizures and abnormal behaviors observed in patients with Lennox–Gastaut syndrome[163]. Cortical neurons of these knock-in mice demonstrated reduced mIPSC amplitude, and treatment with antiepileptic drugs improved the seizure phenotype. *Gabrb3*^+/N328D^ mice showed spontaneous seizures and signs of cognitive impairment, as well as reduced β3 subunit expression in the cerebellum, hippocampus, and thalamus[164]. In addition to α1 and β3 subunits, knock-in mice carrying disease-associated variants in the γ2 subunit have also been developed. Mice heterozygous for γ2(R82Q) variation in GABA_A_R demonstrated behavioral arrest and clear spike and wave discharges on an electroencephalogram akin to the absence and febrile seizure phenotype observed in patients harboring this variant [165,166]. Consistently, they observed reduced cell surface expression of γ2(R82Q) and decreased GABA_A_R-mediated synaptic currents. Additionally, *Gabrg2*^+*/Q390X*^ knock-in mouse displayed intracellular aggregation of γ2(Q390X) subunit, leading to impaired inhibitory GABAergic signaling and neurodegeneration[167]. All these studies demonstrated the utility of mouse models to model the epileptic disease phenotype. However, while many studies focused on homozygous or heterozygous knockout of a major GABA_A_R subunit (i.e., *Gabra1*, *Gabrb2*, *Gabrg2*) in mice[168], relatively few studies explored the effect of a disease-associated variant in vivo. Future studies using heterozygous knock-in mice with specific variants in the GABA_A_R subunits have the potential to enable the exploration of behavioral effects, investigation of synaptic function, and evaluation of drug actions, all of which can drive advancement in clinical discovery.

## 4. Conclusion and outlook

In this review, we have summarized key players in the GABA_A_R proteostasis network that regulate the biogenesis, expression, and function of GABA_A_Rs, including folding, assembly, trafficking, degradation, and synaptic clustering (Fig. 2, Table 1). Significant scientific advancement has shed light on the complex proteostasis network modulating the biogenesis of GABA_A_Rs. Quantitative proteomics is an invaluable tool for discovering novel proteins that interact with GABA_A_Rs and GABA_C_Rs [16,169]. Proteomics studies using human HEK293T cells identified GABA_A_R-interacting proteins, including molecular chaperones (i.e., Hsp90s, Hsp70s, Hsp40s, calnexin, and Hsp47) and ubiquitin-dependent degradation factors (i.e., UBA1, UBR5, UBE3C, SEL1L, and VCP). These high-throughput studies also found that GABA_A_R interactors include: glycosylation enzymes (DDOST, RPN1, RPN2, DPM1, GANAB, and UDP-glucose glycoprotein glucosyltransferase 1 [UGGT1]), translocon-related proteins (SSR1 and signal recognition particle 68 [SRP68]), a COPII subunit (SEC16A), COPI subunits (COPA and COPG2), an endocytosis-related protein (CLINT1), and numerous other proteolysis-related proteins such as ERAD factors. However, the significance of these interactions needs to be further validated in future experiments. Overall, the biogenesis of GABA_A_R relies on the orchestration of various cellular compartments and proteostasis partners to achieve productive folding and assembly and the creation of a mature, functional receptor on the surface.

Additionally, we highlighted pathogenic variants in GABA_A_Rs, particularly epilepsy-associated variants that result in receptor dysfunction, and their known underlying disease-causing molecular mechanism (Fig. 3, Table 2). Many GABA_A_R variants are resistant to current anti-seizure drug treatment. Since the major disease-causing mechanism for GABA_A_R variants is that they fail to reach the plasma membrane for their function, current anti-seizure drugs only work on the surface receptors and thus cannot rescue the function of such trafficking-deficient receptor variants. Therefore, we provided current insights into pharmacological strategies, including receptor-specific PCs and general PRs, to target epilepsy-associated GABA_A_R variants to restore their trafficking and function for disease intervention (Fig. 4). With the development of high-throughput techniques, such as turn-on fluorescent imaging probes that can quantitatively evaluate ligandreceptor interactions, it is feasible to discover new allosteric modulators of GABA_A_R that can regulate receptor function, creating potentials for the development of more efficacious anti-epilepsy drugs[170].

Understanding the molecular mechanisms underlying genetic epilepsy is critical for the development of innovative therapeutic strategies to effectively target epilepsy-associated GABA_A_R variants. While many studies focusing on GABA_A_R variants provided valuable insights into how select variants impact channel gating and activity, much remains to be done to comprehensively characterize the effect of GABA_A_R variants in the early proteostasis regulatory pathway. The development of iPSCs and mouse models for pathogenic GABA_A_R variants, with the aid of recent technological advancements such as super-resolution imaging, has greatly expanded our assets to advance our understanding of GABA_A_R biogenesis, function, and regulation. These models provided physiologically relevant context to study how these variants contribute to disease phenotypes both in vitro and in vivo. This knowledge will be tremendously helpful for designing novel therapeutic approaches to rescue the function of GABA_A_R in patients carrying these devastating disease-causing variants.

## Figures and Tables

**Table 1:** GABA_A_R-interacting proteins that play a key role in regulating receptor proteostasis.

Protein Name	Gene Name	Functional Effect for GABA_A_ receptors	References
BiP/Grp78	HSPA5	↑ folding	[18,19]
Calnexin	CANX	↑ folding	[21]
Calreticulin	CALR	↑ folding	[21]
Hsp47	SERPINH1	↑ assembly	[30]
NACHO	TMEM35A	↑ assembly	[33]
EMC3 & EMC6	EMC3 & EMC6	↑ TM insertion	[31,32]
Grp94	HSP90B1	↑ ERAD	[35]
OS9	OS9	↑ ERAD	[35]
Hrd1/synoviolin	HRD1/SYVN1	↑ ERAD	[35,38]
RNF34	RNF34	↑ ERAD	[39]
TRIM9	TRIM9	↑ ERAD	[40]
Plic–1	UBQLN1	↓ ubiquitination & ERAD, ↑ membrane insertion & surface trafficking	[42,43]
GODZ	ZDHHC3	↑ palmitoylation, ↑ surface trafficking	[57-59]
GABARAP	GABARAP	↑ surface trafficking	[60-63]
NSF	NSF	↑ surface trafficking	[63]
BIG1/2	ARFGEF1/2	↑ surface trafficking	[65,66]
PRIP1/2	PLCL1/2	↑ surface trafficking by regulating phosphorylation	[67,68]
GRIF1/TRAK2	GRIF1/TRAK2	↑ surface trafficking	[75,76]
KIF5A/B/C	KIF5A/B/C	↑ surface trafficking	[79]
HAP1	HAP1	↑ surface trafficking, receptor recycling	[78,110]
Maf	MAF	↑ surface expression	[171]
Macoco	CEP112	↑ surface expression	[171]
Clptm1	CLPTM1	↓ surface trafficking	[53-56]
Gephyrin	GPHN	↑ synaptic clustering	[80-85, 87, 89]
Collybistin	ARHGEF9	↑ synaptic clustering	[86]
Neuroligin2	NLGN2	↑ synaptic clustering	[93]
Neurexin	NRXN	↑ synaptic clustering	[94]
Radixin	RDX	↑ synaptic anchoring for extrasynaptic receptors	[90,172]
GARLH	LHFPL4	↑ synaptic clustering	[95]
Neurobeachin	NBEA	↑ synaptic surface expression	[97]
Shisa7	SHISA7	↑ trafficking & surface expression, regulate channel gating	[98,100, 101]
TMEM132B	TMEM132B	↑ surface expression	[102]
AP2	AP2A1/AP2A2/AP2M1/AP2B1	↑ receptor endocytosis	[108]
Neuroplastin	NPTN	↓ receptor endocytosis, ↑ synaptic/extrasynaptic anchoring	[173,174]
Ankyrin3	ANK3	↓ receptor endocytosis, ↑ synaptic stability	[111,175]
GIT1	GIT1	↑ synaptic surface stability & synaptic strength	[176]
βPIX	ARHGEF7	↑ synaptic surface stability & synaptic strength	[176]

↑, increased; ↓, decreased.

**Table 2:** GABA_A_R variants that result in defective receptor function.

Subunit and variant	Variation type	Structural region	Associated disease	Functional impairment	References
α1(S76R)	Missense	NTD	DS	↓ total and surface expression, ↓ GABA-evoked currents	[147,177]
α1(F104C)	Missense	NTD	JME	↓ GABA-evoked currents	[177]
α1(R112Q)	Missense	NTD	EOEE; DS/IS	↓ GABA potency	[178]
α1(N115D)	Missense	NTD	EOEE	↓ GABA potency	[178]
α1(R214H/C)	Missense	NTD	DS	↓ GABA-evoked current amplitude, ↓ total and surface expression, ↑ subunit misfolding, ↑ERAD	[147,177,179,180]
α1(L215P)	Missense	NTD	DS	↓ GABA-evoked current amplitude, ↓ desensitization rate, ↓ activation rate, ↑ deactivation rate	[180]
α1(D219N)	Missense	NTD	JME	↓ total and surface expression, ↓ GABA-evoked currents, ↑ aggregation propensity, ↑ ERAD, ↑desensitization rate	[21,147,181]
α1(G251D)	Missense	TM1	EE	↓ surface expression, ↑ aggregation propensity, ↑ subunit oligomerization, ↓ GABA-evoked currents	[147,177]
α1(P260L/S)	Missense	TM1	OS/WS	↓ total and surface expression, ↓ receptor assembly, ↓ GABA-evoked currents	[147,182]
α1(M263T)	Missense	TM1	WS	↓ surface expression, ↓ receptor assembly, ↓ GABA-evoked currents	[147,182]
α1(F272fs287X)	Frameshift	TM1	EE	↓ surface expression, ↑ ER retention, ↑ subunit oligomerization, ↓ GABA-evoked currents	[183]
α1(V287I)	Missense	TM2	CAE/DS	↓ GABA-evoked currents, ↓ desensitization rate	[180]
α1(T289P)	Missense	TM2	EOEE	↓ GABA-evoked currents	[147,177]
α1(V290fs299X)	Frameshift	TM2	EE	↓ surface expression, ↑ ER retention, ↑ subunit oligomerization, ↓ GABA-evoked currents	[183]
α1(T292I)	Missense	TM2	WS/LGS	↓ surface expression, ↓ GABA-evoked currents, ↓ GABA sensitivity	[184]
α1(L296S)	Missense	TM2	WS	↓ total and surface expression, ↓ GABA-evoked currents, ↑ GABA potency, ↑ Zn^2+^ sensitivity	[178]
α1(K306T)	Missense	TM2–3 loop	MAE-like/DS	↓ GABA-evoked currents	[177]
α1(W315L)	Missense	TM3	WS	↓ total and surface expression, ↓ GABA-evoked currents, ↑ GABA potency, ↑ Zn^2+^ sensitivity	[178]
α1(A322D)	Missense	TM3	JME	↓ total and surface expression, ↓ peak GABA-evoked currents, ↓ subunit folding, ↑ ERAD	[147,185,186]
α1(S326fs328X)	Frameshift	TM3	CAE	mRNA non-sense mediated decay (NMD) and ↑ ERAD of mutant protein	[187]
α1(A332V)	Missense	TM3	EOEE	↑ GABA potency, ↑ desensitization rate	[188]
α1(K401fs425X)	Frameshift	TM3–4 loop	EE	↓ surface expression, ↑ ER retention, ↓ GABA-evoked currents	[183,189]
β2(M79T)	Missense	NTD	EE	↓ total and surface expression	[190]
β2(Y181F)	Missense	NTD	DS	↓ desensitization rate, ↓ activation rate, ↑ deactivation rate	[180]
β2(Q209F210delinsH)	Frameshift/Indel	NTD	ID	↓ total and surface expression, ↓ peak GABA-evoked currents, ↓ protein stability, ↓ trafficking, ↑ ERAD	[191]
β2(F224C)	Missense	NTD	EE	↓ total and surface expression, ↑ GABA potency	[190]
β2(R240T)	Missense	NTD	AS/ES	↓ total and surface expression, ↓ peak GABA-evoked currents, ↓ protein stability, ↓ trafficking, ↑ ERAD	[128] [191]
β2(F245S)	Missense	TM1	EE	↓ total and surface expression, ↑ GABA potency	[190]
β2(I246T)	Missense	TM1	LGS-like/EME	↓ total and surface expression, ↓ trafficking, ↓ GABA-evoked currents, ↑ GABA potency at low [GABA]	[192] [191]
β2(P252L)	Missense	TM1	EOEE/IS/LGS-like	↓ GABA-evoked currents	[192]
β2(V282A)	Missense	TM2	Chorea, CVI	↓ GABA-evoked currents, ↑ GABA potency at low [GABA]	[192]
β2(T287P)	Missense	TM2	EME	↓ total and surface expression, ↓ GABA-evoked currents, ↑ intracellular retention	[193]
β2(I288S)	Missense	TM2	LGS-like	↓ total and surface expression, ↓ GABA-evoked currents	[190,192]
β2(I299S)	Missense	TM2–3 loop	IS	↓ GABA-evoked currents	[192] [191]
β2(V302M)	Missense	TM2–3 loop	EE	↓ total and surface expression, ↑ GABA potency	[190]
β2(K303N)	Missense	TM2–3 loop	EE	↓ total and surface expression, ↑ GABA potency	[190]
β2(F331del)	Frameshift	TM3–4 loop (ICD)	DS	↓ GABA-evoked currents, ↓ desensitization rate, ↓ deactivation rate	[180]
β3(S76C)	Missense	NTD	FS/MAE	↓ GABA-evoked currents, ↓ GABA potency	[140]
β3(E77K)	Missense	NTD	WS	↓ GABA-evoked currents, ↑ GABA potency	[140]
β3(M80K)	Missense	NTD	FS/T	↓ GABA-evoked currents, ↓ GABA potency	[140]
β3(M80L)	Missense	NTD	EE	↓ GABA potency	[140,190]
β3(N110D)	Missense	NTD	WS/IS	↓ single channel burst duration	[194]
β3(D120N)	Missense	NTD	LGS	↓ GABA-evoked currents, ↓ GABA potency	[194]
β3(K127R)	Missense	NTD	MAE	↓ surface expression, ↓ GABA-evoked currents, ↓ GABA potency	[140,190]
β3(L165Q)	Missense	NTD	FS	↓ GABA-evoked currents	[140]
β3(L170R)	Missense	NTD (cys-loop)	EOEE	↓ surface expression, ↓ receptor trafficking, ↑ ER retention, ↓ GABA-evoked peak currents, ↑ GABA potency, ↑ desensitization rate, ↓ activation rate, ↓ deactivation rate, ↓ single channel open probability, ↓ mean open time, ↓ burst duration	[195]
β3(E180G)	Missense	NTD	LGS	↓ GABA-evoked currents, ↓ GABA potency, spontaneous channel openings	[140,194]
β3(R232Q)	Missense	NTD	EE	↓ surface expression	[190]
β3(Y245H)	Missense	TM1	EE	↑ GABA potency	[190]
β3(L278F)	Missense	TM2	EE	↓ surface expression, ↓ GABA potency	[190]
β3(T281I)	Missense	TM2	OS	↓ total and surface expression	[190]
β3(L284P)	Missense	TM2	ES	↓ GABA-evoked currents, ↑ GABA potency	[140]
β3(T287I)	Missense	TM2	OS	↑ GABA potency	[140,190]
β3(T288N)	Missense	TM2	EOEE	↓ GABA-evoked currents, ↓ GABA potency, ↑ activation rate, ↓ single channel open probability, ↓ mean open time, ↓ burst duration	[195]
β3(I300T)	Missense	TM2–3 loop	Myo	↓ GABA-evoked currents, ↑ GABA potency	[140]
β3(P301L)	Missense	TM2–3 loop	FS/Myo	↓ GABA-evoked currents, ↓ GABA potency	[140]
β3(Y302C)	Missense	TM2–3 loop (ECD)	LGS	↓ GABA-evoked currents, ↓ GABA potency, ↑ deactivation rate	[140,194]
β3(A305V)	Missense	TM3	EOEE	↓ surface expression, ↓ receptor trafficking, ↑ ER retention, ↓ GABA-evoked peak currents, ↑ GABA potency, ↑ desensitization rate, ↑ Zn^2+^ inhibition, ↓ activation rate, ↓ deactivation rate, ↓ single channel open probability, ↑ mean open time, ↑ burst duration	[140,195]
β3(N328D)	Missense	TM3	LGS	↓ surface expression, ↓ postsynaptic clustering, ↓ GABA-evoked currents	[196]
β3(E357K)	Missense	TM3–4 loop (ICD)	JAE	↓ surface expression, ↓ postsynaptic clustering, ↓ GABA-evoked currents	[196]
γ2(Q40X)	Nonsense	NTD	DS	↓ surface expression, NMD	[197]
γ2(N79S)	Missense	NTD	GES	↓ receptor assembly, ↓ surface expression, ↓ trafficking	[198]
γ2(R82Q)	Missense	NTD	CAE/FS	↓ receptor assembly, ↓ surface expression, ↑ ER retention, ↓ BZD sensitivity	[138, 166, 198-200]
γ2(P83S)	Missense	NTD	GES	↓ receptor assembly, ↓ surface expression, ↑ ER retention	[198]
γ2(T90R)	Missense	NTD	DS	↓ surface expression, ↓ GABA-evoked currents, ↓ receptor assembly, ↑ ER retention	[180]
γ2(A106T)	Missense	NTD	Unclassified EE	↓ surface expression, ↓ GABA-evoked currents, ↓ desensitization rate, ↑ activation rate, ↓ deactivation rate, ↓ GABA potency	[201]
γ2(I107T)	Missense	NTD	Unclassified EE	↓ surface expression, ↓ GABA-evoked currents, ↑ Zn^2+^ sensitivity, ↑ ER retention, ↓ deactivation rate, ↓ GABA potency	[201]
γ2(R136X)	Nonsense	NTD	FS/GEFS+	↑ ER retention, ↓ total expression, ↓ trafficking, NMD	[197,202]
γ2(R177G)	Missense	NTD	FS	↓ BZD sensitivity, ↓ glycosylation, ↓ trafficking, ↓ surface expression	[137]
γ2(G257R)	Missense	NTD	RE	↓ trafficking, ↓ surface expression, ↓ postsynaptic clustering, ↓ palmitoylation, ↑ ER retention	[203]
γ2(P282S)	Missense	TM1	Unclassified EE	↓ surface expression, ↓ GABA-evoked currents, ↑ Zn^2+^ sensitivity, ↑ ER retention, ↓ deactivation rate	[201]
γ2(P302L)	Missense	TM2	DS	↓ surface expression, ↓ GABA-evoked currents, ↓ GABA potency, ↑ desensitization rate	[204]
γ2(R323W)	Missense	TM2	Unclassified EE	↓ surface expression, ↓ GABA-evoked currents, ↑ Zn^2+^ sensitivity, ↓ desensitization rate, ↑ deactivation rate, ↓ GABA potency	[201]
γ2(R323Q)	Missense	TM2	Unclassified EE/RE	↓ surface expression, ↓ GABA-evoked currents, ↑ Zn^2+^ sensitivity, ↓ activation rate, ↑ deactivation rate, ↓ GABA potency	[201,203]
γ2(K328M)	Missense	TM2–3 loop	FS/GEFS+	↓ GABA-evoked currents, ↑ deactivation rate	[205-207]
γ2(F343L)	Missense	TM3	Unclassified EE	↓ surface expression, ↓ GABA-evoked currents, ↑ activation rate, ↓ deactivation rate	[201]
γ2(Q390X)	Nonsense	TM3–4 loop (ICD)	GEFS+/DS	↓ surface expression, ↓ GABA-evoked currents, ↓ trafficking, ↑ aggregation, ↑ ER retention, ↑ polyubiquitination	[197]
γ2(W429X)	Nonsense	TM3–4 loop (ICD)	FS/GEFS+	↓ total and surface expression, ↓ GABA-evoked currents, ↓ trafficking, ↑ ER retention, ↑ polyubiquitination	[197,208]
γ2(S443delC)	Frameshift	TM3–4 loop (ICD)	FS/GEFS+	↓ total and surface expression, ↑ ER retention, ↓ GABA-evoked currents, ↑ Zn^2+^ sensitivity	[209]

AS, Atonic seizure; CAE, childhood absence epilepsy; CVI, cortical visual impairment; DS, Dravet syndrome; EE, epileptic encephalopathy; EME, early myoclonic encephalopathy; EOEE: early onset epileptic encephalopathy; ES, epileptic spasm; FS, febrile seizure; GES, genetic epilepsy syndrome; GEFS+ , Genetic epilepsy syndrome with febrile seizures plus; ID, intellectual disability; IGE, idiopathic generalized epilepsies; IS, infantile spasms; JAE, juvenile absence epilepsy; JME, juvenile myoclonic epilepsy; LGS, Lennox-Gastaut syndrome; MAE, epilepsy with myoclonic-atonic seizures; Myo, myoclonic; RE, rolandic epilepsy; T, tonic; WS, West syndrome; NTD, N-terminal domain; TM, transmembrane; BZD, benzodiazepine; ↑, increased; ↓, decreased.

## Data Availability

This review does not contain original research data.

## Abbreviations:

5-HT_3_R: 5-hydroxytryptamine type-3 receptor
AKAP: A-kinase anchor protein
ANK3: giant ankyrin-G
AP2: adaptor protein 2
ATF6: activating transcription factor 6
ATG8: autophagy-related protein 8
BIG: brefeldin A-inhibited guanine nucleotide-exchange factor
BiP: binding immunoglobulin protein
CAE: childhood absence epilepsy
CaMKII: Ca^2 +^/calmodulin-dependent protein kinase II
CHOP: C/EBP homologous protein
Clptm1: Cleft lip and palate transmembrane protein 1
CNS: central nervous system
Cryo-EM: cryo-electron microscopy
DKO: double knock out
ECD: extracellular domain
EMC: ER membrane protein complex
ER: endoplasmic reticulum
ERAD: ER-associated degradation
GABA_A_Rs: γ-aminobutyric acid type A receptors
GABARAP: GABA_A_-receptor-associated protein
GARLH: GABA receptor-like homolog
GC: glucocerebrosidase
GlyR: glycine receptor
GODZ: Golgi-specific DHHC zinc finger protein
GRIF-1: GABA_A_R-interacting factor 1
Grp94: glucose-regulated protein 94
HAP1: huntingtin-associated protein 1
Hrd1: HMG-CoA 3-hydroxy-3-methylglutaryl-coenzyme A reductase degradation 1
iPSC: induced pluripotent stem cell
IRE1: inositol-requiring enzyme 1
JME: juvenile myoclonic epilepsy
KIF5: kinesin-related protein 5
KO: knockout
LIR: LC3-interacting region
LMAN1: lectin mannose-binding 1
LTD: long-term depression
LTP: long-term potentiation
mIPSCs: miniature inhibitory post-synaptic currents
nAChR: nicotinic acetylcholine receptor
NAM: negative allosteric modulator
NMD: nonsense-mediated mRNA decay
NSF: *N*-ethylmaleimide-sensitive factor
NTD: N-terminal domain
PAM: positive allosteric modulator
PC: pharmacological chaperone
PERK: protein kinase RNA-like endoplasmic reticulum kinase
PDI: protein disulfide isomerase
PIP2: phosphatidylinositol 4,5-bisphosphate
PKA: protein kinase A
PKC: protein kinase C
PKG: protein kinase G
PP1α: protein phosphatase 1α
PR: proteostasis regulator
PRIP: phospholipase C-related catalytically inactive protein
RNF34: ring finger protein 34
TM: transmembrane
UPR: unfolded protein response
UPS: ubiquitin-proteasome system
VCP: valosin-containing protein
WT: wild type
Xbp1: X-box binding protein 1
